# Park morphology and urban structure for active living: a suburban case from Seongnam City

**DOI:** 10.3389/fpubh.2026.1744227

**Published:** 2026-01-23

**Authors:** Kitae Park, Jeongwoo Lee, Yesong Shin

**Affiliations:** 1 Department of Landscape Architecture and Urban Planning, Texas A&M University, College Station, TX, United States; 2 Department of Urban Design and Studies, Chung-Ang University, Seoul, Republic of Korea

**Keywords:** built environment, environmental justice, greenspace equity, park morphology, physical activity, public health, suburban planning

## Abstract

**Introduction:**

This study investigates how park morphology and built environments are associated with residents’ physical activity in a suburban context, focusing on Seongnam City, South Korea. It further examines how these relationships differ between master-planned new towns and incrementally developed old towns, which reflect contrasting planning legacies and spatial structures.

**Methods:**

Using high-resolution spatial data and geocoded survey responses from 577 residents, the analysis incorporates morphological indicators—including edge complexity and pedestrian connectivity—alongside physical activity data from the International Physical Activity Questionnaire. Binary logistic regression models were applied separately for old and new towns to examine associations between park morphology and physical activity, controlling for sociodemographic, health, and perceptual variables.

**Results:**

The findings reveal marked spatial and behavioral inequities. Although old towns contain a larger total area of neighborhood parks, their location on steep slopes or at the urban fringe, combined with fragmented distribution of other park types, limits accessibility and routine use. Residents in old towns visit parks less frequently, rely more on vehicles for access, and report lower satisfaction with neighborhood and park environments. In contrast, new towns feature an interwoven green network with centrally located waterfront parks and evenly distributed small parks within residential superblocks, supporting higher accessibility, stronger user preference, and more frequent daily park use. In new towns, higher edge density and greater neighborhood park area were significantly associated with physical activity, corresponding to a 7% increase and tenfold higher odds of participation, respectively—associations not observed in old towns.

**Discussion:**

These results underscore that green space equity extends beyond mere provision to include spatial integration and usability. Promoting health through urban green space requires context-sensitive planning strategies that ensure equitable access and functional design across different neighborhood contexts.

## 1 Introduction

Urban green spaces are recognized for their ecological and social value, as they contribute to environmental quality and public health. Alongside ecological benefits such as air purification and heat mitigation, green spaces support physical health through physical activity (PA), mental well-being, and social interaction (1–5). However, these benefits are not determined solely by the presence of green space. Increasing attention has been placed on the importance of equitable access, spatial integration, and functional design in shaping how people interact with and benefit from these environments.

A growing body of research emphasizes that accessibility, rather than simple availability, is central to achieving environmental justice in urban settings (6–9). Structural barriers such as limited park proximity, poor pedestrian infrastructure, and perceived or actual safety concerns disproportionately affect low-income and minority populations, reducing their ability to use green spaces effectively (10–12). Furthermore, disparities often extend to park quality, with parks in disadvantaged communities more likely to be under-maintained and lacking in diverse recreational amenities, further limiting their public health impact (13–15).

These disparities are evident in suburban South Korea, specifically in the contrast between master-planned new towns and incrementally developed older neighborhoods. National planning guidelines mandate a minimum amount of green space per capita, but suburban implementation varies considerably. Master-planned new towns are large-scale and feature continuous green corridors and pedestrian-oriented layouts (16). By contrast, older suburban areas that developed incrementally feature fragmented parks with limited connectivity (17, 18). Larger parks are often located along the old town’s mountainous edges, reducing their accessibility for daily use. In these areas, residents experience overlapping disadvantages, including limited access to a park, weak pedestrian infrastructure, and greater concerns of traffic hazards and personal safety (19).

Quantitative assessments of the relationship between green space and health outcomes increasingly integrate objective environmental metrics with behavioral data. While traditional approaches have relied on spatial analysis and basic quantitative techniques to evaluate green space accessibility and exposure, they often fall short in capturing fine-grained environmental characteristics. Recent advances in high-resolution remote sensing technologies address this gap by enabling more precise measurement of vegetation density, land use patterns, and spatial connectivity (20–23). Among these spatial features, park morphology or the design and structure of green spaces, is critical to shaping PA-related behaviors. Park size, configuration, vegetation type, and recreational amenities—trails and playgrounds—influence the type, duration, and frequency of PA (24–27). Perceived safety, lighting, visibility, and social presence also affect the use of green spaces, particularly among vulnerable groups (28, 29).

Several dependent variables are used to assess the health benefits of urban green spaces, with PA being the most frequently examined. Common indicators include the total duration of moderate-to-vigorous activity, frequency of walking or exercise, and whether individuals meet the recommended activity guidelines. Accelerometers or standardized instruments such as the International PA Questionnaire (IPAQ) are used to measure these outcomes. Prior studies have shown that people living close to parks meet the recommended PA guidelines and report higher frequencies of walking, running, and recreational exercise, as captured by both accelerometer data and IPAQ responses (30, 31). However, these findings are mixed, as park access is often moderated by factors such as park quality, built environment (BE), and user perception and characteristics (32, 33).

Recognizing the combined influence of park accessibility, morphology, and neighborhood context is essential for developing spatial strategies to promote equitable physical health. In this study, PA is treated as a behavioral indicator of physical health, and differences in PA participation are interpreted as a reflection of health equity across spatial and social contexts. Planning efforts must move beyond simply increasing park area and focus on addressing access barriers, improving design, and creating inclusive environments that encourage PA (34–36). However, few studies have jointly examined park morphology and BE within an integrated framework, particularly in suburban contexts where spatial structures vary. In South Korea, suburban areas where new and old towns reflect distinct planning legacies, provide a unique opportunity to explore how environmental inequalities in urban form translate into behavioral and health disparities through differences in park access and PA.

To address these gaps, this study aims to answer the following three specific research questions. First, what is the influence of park morphology and BE characteristics on resident’s PA levels within this suburban context, utilizing high-resolution spatial indicators and a validate PA instrument? Second, how do contrasting planning legacies and spatial structures, specifically comparing master-planned new towns with incrementally developed old towns determine residents’ opportunities for active living? Third, how do the interactions among planning legacies, spatial structures, and sociodemographic factors produce disparities in park use and PA within this single suburban city, thereby highlighting the needs for physical design considerations, public health, and environmental justice?

High-resolution spatial data are used to assess detailed morphological indicators at the level of individual residential locations, including park connectivity, patch density, edge complexity, and proximity to nearby green spaces. These spatial variables are linked to individual PA data collected from 577 residents using the IPAQ-Short Form (SF). Binary logistic regression models are employed to analyze the association between BE characteristics and PA, while controlling for sociodemographic, health, and perceptual factors.

## 2 Materials and methods

### 2.1 Study area

**Figure 1:**
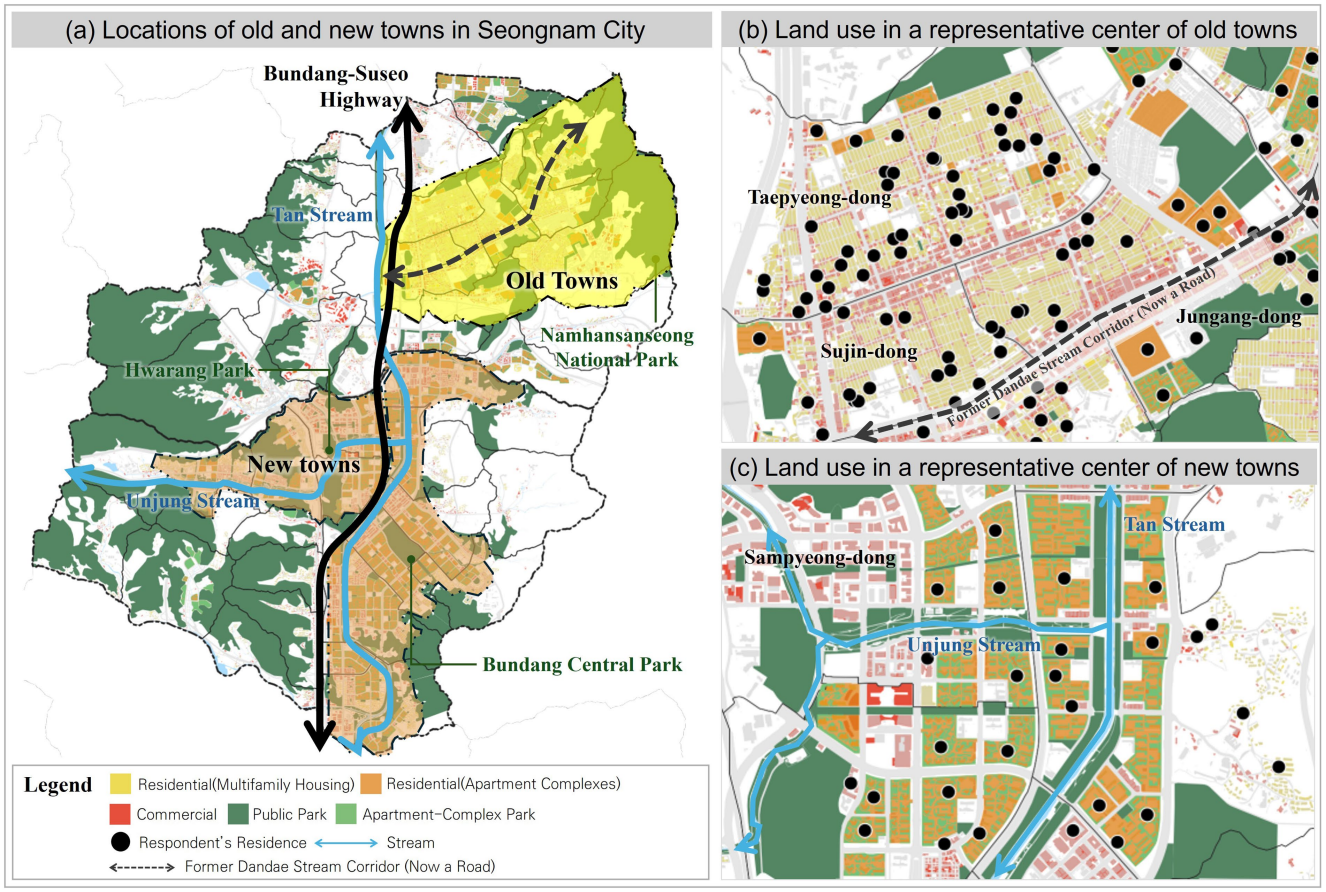
Spatial distribution of old and new towns and representative land-use patterns in Seongnam City. **(a)** Locations of old and new towns in Seongnam City. **(b)** Land use in a representative center of old towns. **(c)** Land use in a representative center of new towns.

This study examines two neighborhoods in Seongnam City, southeast of Seoul, that reflect contrasting urban development trajectories. The old towns, formed in the 1970s as resettlement areas for low-income populations, occupy hilly terrain and are marked by irregular street patterns, narrow alleys, and dense clusters of multi-family housing. Incremental development in these areas has led to fragmented land use and limited public amenities. In contrast, the new towns, master-planned during the 1990s and 2000s, feature structured superblocks with high-rise apartments, integrated pedestrian networks, wide roads, and clearly delineated land uses that separate residential and commercial zones. The Tan Stream runs through both areas, serving as a shared ecological and recreational corridor, but the differing urban forms and planning histories illustrate a broader transition from infrastructure-driven growth to comprehensive suburban urbanization (Figure 1a).

In old towns, the absence of planned green spaces is notable. Existing green areas primarily include the steeply sloped Namhansanseong National Park and a few neighborhood parks embedded in surrounding hillsides. The challenging topography limits everyday accessibility, making these areas more conducive to recreational hiking and biking than to routine use. Although Dandae Stream once flowed through the old town center, it was covered during 1990s redevelopment and replaced by a roadway. Consequently, access to waterfront green space is constrained, with the only remaining park located at the neighborhood’s fringe (Figure 1b).

By contrast, the new town features an interwoven green network connected to both the Tan Stream and the Unjung Stream, which flow through the center of the area. This network links seamlessly with neighborhood and small parks, extending into residents’ daily activity zones. Originating from residential clusters, these green corridors enhance neighborhood walkability and facilitate routine engagement with green spaces (Figure 1c).

### 2.2 Research design

A comparative case study design was used to investigate the complex relationships between green space accessibility, BE characteristics, and residents’ PA levels in the two distinct urban areas of Seongnam City. The contrasting planning frameworks and spatial structures of the old and new town areas offer valuable opportunities for comparison, making them suitable for investigating the relationship between urban form and health behaviors.

To assess this relationship, individual-level data were collected from residents of both old and new towns, yielding a final sample of 577 respondents, comprising 253 from old towns and 324 from new towns. A standardized self-reporting instrument was used to measure PA, categorized into binary variables to distinguish between physical inactivity and any level of activity.

The unit of analysis for environmental exposure was defined as a 400-m pedestrian network buffer around each respondent’s residence. This scale has been widely adopted in physical activity research to capture meaningful neighborhood-scale exposure. Theoretically, a 400-m buffer represents an approximately 5-to-10-min walk at average human speeds, which is recognized in urban planning and public health literature as the effective neighborhood where residents are most frequently influenced by built environment features (37–39). To ensure the appropriateness of this scale within the large-scale superblocks of New Towns, we conducted a sensitivity analysis testing distances from 400 m to 800 m. The results demonstrated a clear distance-decay pattern: although the positive associations between park morphology and physical activity remained statistically significant across all tested distances up to 800 m, the strength of the correlation notably diminished as the buffer size increased. The association reached its highest magnitude and statistical significance specifically at the 400-m range (Coef = 0.097, *p* = 0.004), compared to the weakest effect observed at 800 m. Therefore, the 400-m buffer was empirically validated as the most sensitive and representative scale for capturing the direct impact of local environmental affordances on habitual walking behavior (Appendix Table S1 and Appendix Figure S1).

Using QGIS, spatial indicators were extracted from each buffer to quantify green space availability, accessibility, and configuration. The analysis was conducted in two stages. First, two-sample t-tests were conducted to compare the sample means and proportions between residents of old and new towns. Second, separate binary logistic regression models were applied to each town type to examine the association between park morphology and PA, controlling for demographic characteristics, self-reported health status, and environmental perceptions. All analyses were performed using IBM SPSS Statistics 27.

### 2.3 Data

#### 2.3.1 Multidimensional assessment of the BE

**Figure 2:**
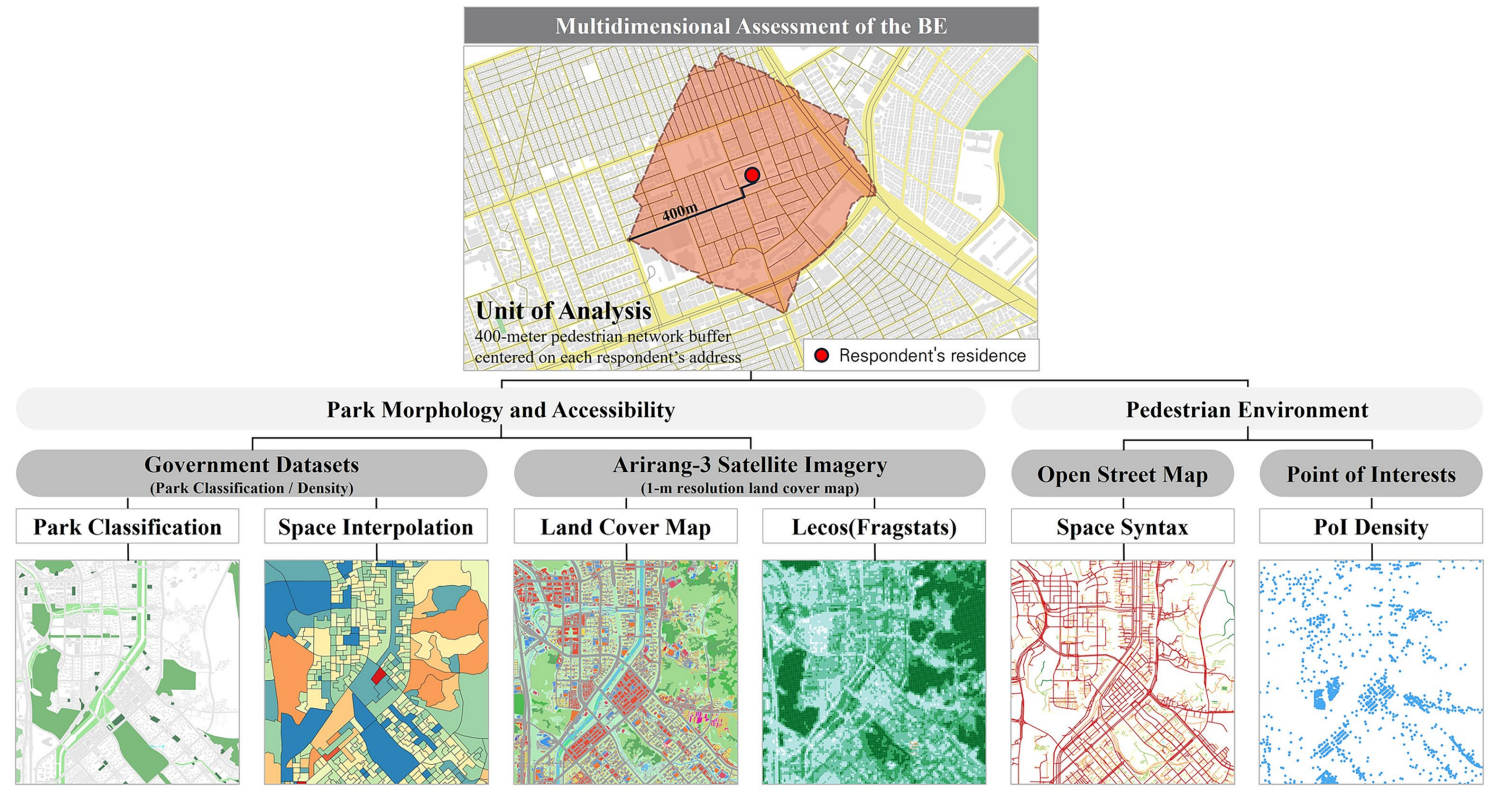
The approach used to measure the multidimensional characteristics of the BE.

To comprehensively assess the BE, this study used multiple datasets capturing park morphology, accessibility, and the pedestrian environment (Figure 2). To ensure spatial precision, official polygon layers representing park boundaries were compiled alongside point layers detailing park attributes, including park type. As government datasets often lack recent boundary updates and newly created green patches, we cross-validated and refined them using two complementary sources: OpenStreetMap (OSM) and a 1-m resolution land cover map layer derived from Arirang-3 satellite imagery. Integrating these three data sources produced a harmonized green space database that minimized topological gaps and captured courtyard vegetation within apartment complexes, as well as small-scale greenery, such as pocket parks.

A combination of spatial and classification data was used to measure the park morphology, accessibility, and type attributes. Park classification data were initially compiled from government sources, which categorize public parks into four types. Neighborhood parks (≥1 ha), small or children’s parks (<1 ha), waterfront parks, and mountain parks. According to the legal standards in Korea, neighborhood parks are defined as core community green spaces within 500 m of a residential area (40). Small parks and children’s parks provide localized recreational opportunities, whereas waterfront parks along the Tan Stream are ecological corridors. Mountain parks offer expansive natural areas for hiking and outdoor adventure activities.

**Figure 3:**
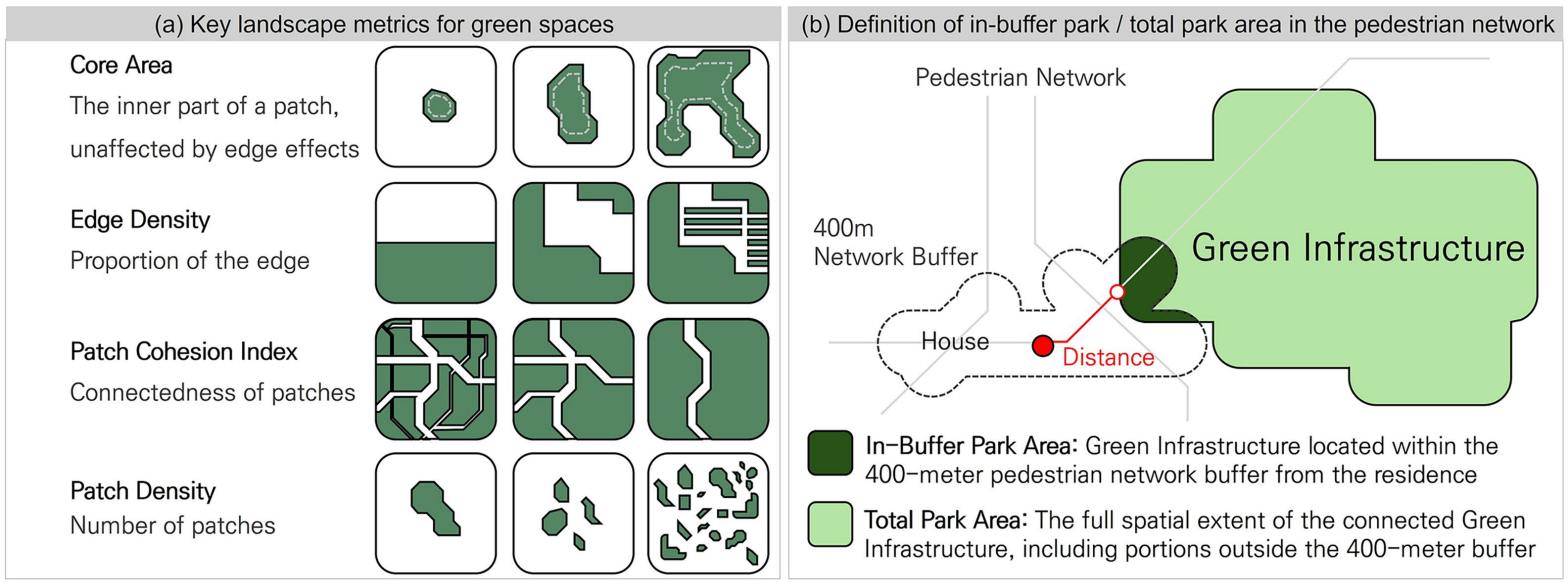
Green space metrics measurement methodology.

Park morphology within each respondent’s 400-m pedestrian network buffer was quantified using landscape metrics generated by the LecoS plugin in QGIS. Four key metrics—Core Area, Edge Density, Patch Density, and Patch Cohesion Index—were used to describe internal continuity, boundary complexity, spatial distribution, and overall cohesion, as they may influence how residents perceive and use nearby parks. We assessed park accessibility by measuring the shortest network-based walking distance from each respondent’s geocoded residence to the nearest park, offering a more realistic indicator of walkability than the Euclidean distance. Total green space availability was estimated as the cumulative area of parks fully or partially intersecting the 400-m buffer, using the AID-PRIGSHARE plugin in QGIS [(41); Figure 3].

The pedestrian environment was evaluated using the OSM-based pedestrian network data, adjusted for slope using Tobler’s walking speed function (42). A Weighted Segment Analysis was conducted using the QGIS Space Syntax Toolkit to derive the Normalized Angular Integration (NAIN) index. While both NAIN (representing destination-oriented accessibility) and Normalized Angular Choice (NACH, representing through-movement potential) were initially considered, NAIN was selected as the primary metric because it demonstrated a more robust and statistically significant association with physical activity in preliminary tests. As a key space syntax metric, NAIN captures the relative accessibility and connectivity of each street segment, serving as a proxy for the pedestrian movement potential within the neighborhood (43, 44). Beyond street networks, facility accessibility was assessed using Points of Interest (POI) data representing retail, healthcare, and recreational services. Counts of each POI category within the 400-m buffer were calculated to reflect the local service environment. Finally, population, housing, and business densities were derived from Korea’s smallest census unit, the *jipgyegu* formula:

$$Z_{k}=\sum_{i=1}^{n}(\frac{A_{i}}{A_{j}}\times Z_{j})$$

where $A_{i}$ is the area of the portion of the *jipgyegu* that falls inside buffer k , $A_{j}$ ​ is the total area of that *jipgyegu*, and $Z_{j}$ is the original census attribute value. These three density indicators were chosen as core metrics for quantifying the compact urban form, which is critical for promoting walkability and active living (45). Their inclusion is for capturing the fundamental structural contrast between the incrementally developed old towns and the master-planned new towns in our comparative analysis.

#### 2.3.2 Survey data

An online questionnaire survey was conducted from May 12 to 18, 2023 for Seongnam City residents aged 15 years and above. A total of 645 respondents completed the survey, providing their residential addresses in road name format, with all responses collected under strict measures to ensure anonymity. The study protocol was approved by the Institutional Review Board of the researcher’s institution (IRB No. 1041078-202110-HR-310-0), and informed consent was obtained prior to the first survey screen. After excluding incomplete responses and addresses outside the study area, the final sample comprised 577 valid responses. Of these, 253 respondents resided in old town neighborhoods and 324 in new town areas. Each geocoded home address was linked to a 400-m pedestrian-network buffer, enabling the integration of individual survey responses with objective indicators of the BE. This integration produced a person–environment matrix, with the variable descriptions summarized in Table 1.

**Table 1:** Description of variables.

Class	Variables	Description	Data source
Respondent’s personal characteristics, environmental perception, and PA levels			
Level of PA	IPAQ-MET	Total weekly PA (sum of walking + moderate + vigorous MET-minutes); recoded to Inactive, Minimally Active, HEPA	Online survey
Demographic and health characteristics	Age	Age grouped as 15–29 (youth), 30–49 (middle-aged), ≥50 (older)	
	Gender	Male/Female	
	Income	Monthly household income bands. < 2 M KRW, 2–5 M KRW, > 5 M KRW	
	Education	Highest educational attainment (high school or below, college)	
	Housing Type	Apartment = 1; Detached/Villa/Other = 0	
	Health Status (EQ-5D)	Mobility, Self-Care, Usual Activities, Pain/Discomfort, and Anxiety/Depression were each rated on a 5-point Likert scale, and the EQ-5D-5L index was calculated using the Korean value set (−0.066 to 1)	
Park utilization	Park Visit Frequency	Categorized as Rare (less than once a month), Occasional (once a week or 1–2 times a month), or Frequent (2–3 times a week or daily)	
Environmental perception	Perceived Neighborhood Environment	5-point Likert scale covering Green/Natural environment, Infrastructure & Public services, Walkability & Safety, Social cohesion	
	Perceived Park Environment	5-point Likert scale covering Location, Facilities, Safety/Maintenance	
BE measures within a 400-m network buffer around each respondent’s residence			
Park morphology and accessibility	Core Area	Interior (non-edge) park area within 400-m network buffer	Ministry of Land, Infrastructure, and Transport’s and Land Cover Map (2022) and Open Street Map
	Edge Density	Edge length of park per unit area within a 400-m network buffer	
	Patch Cohesion Index	Connectivity of park patches within a 400-m network buffer	
	Patch Density	Number of park patches per unit area within a 400-m network buffer	
	Park Proximity	Network distance to nearest park (by park type)	
	Total Park Area	Sum of all park polygons touching 400-m network buffer (by park type)	
	In-Buffer Park Area	Park area entirely inside 400-m network buffer (by park type)	
Pedestrian environment	Municipal Typology	New town = 1; Old town = 0	Online Survey
	Population Density	Population density within a 400-m network buffer from home	SGIS 2022
	Household Density	Household density within a 400-m network buffer from home	
	Housing Density	Housing density within a 400-m network buffer from home	
	Facility Accessibility	Poi Counts within buffer. Transport facilities, Shopping centers, Healthcare, Sports services, Pubs & Accommodation, Community facilities, Retail facilities, Schools	Ministry of Land, Infrastructure, and Transport’s and Gyeonggi Data Dream
	Street Design	Mean Normalized Angular Integration for street segments in buffer (space-syntax walkability)	Open Street Map
	Slope	Slope level within a 400-m network buffer from home and the entire area of old and new towns	Ministry of Land, Infrastructure, and Transport’s

The questionnaire included four thematic sections designed to capture PA, park use and perceptions, neighborhood satisfaction, and individual background characteristics. PA was assessed using the IPAQ-SF, and respondents were asked to report the duration (i.e., number of minutes) spent walking and engaging in moderate and vigorous activities during a typical week. These responses were converted into weekly metabolic equivalent (MET) minutes, and activity levels were classified per the IPAQ scoring protocol as inactive, minimally active, or health-enhancing PA (HEPA) (46).

Park usage was assessed based on visit frequency, with respondents categorized as rare users (less than once a month), occasional users (once a week or one to two times a month), or frequent users (two to three times a week or daily). Respondents rated their satisfaction with park location, facilities, and safety or maintenance using a 5-point Likert scale. To complement these items, neighborhood perception was measured using the same scale, capturing satisfaction with greenery and natural features, infrastructure and public services, walkability and safety, and social cohesion. Collectively, these variables offer a multidimensional perspective on how residents perceive and interact with their local environment.

Environmental perceptions were analyzed alongside sociodemographic and health-related characteristics. The collected information included age group, gender, monthly household income, educational attainment, and housing type. Self-rated health was measured using the 5-level EQ-5D version (EQ-5D-5L) instrument, which evaluates five domains: mobility, self-care, usual activities, pain/discomfort, and anxiety/depression. The scores were converted into an index using the South Korean value set ranging from −0.066 to 1, with higher values indicating better perceived health (47).

## 3 Results

### 3.1 Comparison of BE and perceived spatial quality between old and new towns

Seongnam City’s old and new towns exhibit distinct urban development patterns shaped by contrasting planning approaches (Table 2). Although both cover similar land areas, old towns accommodate nearly twice the population of new towns, reflecting the dominance of compact, low-rise multi-family housing on small plots. This dense development has resulted in a highly compact urban form with limited open space. Many of these dwellings are situated on sloped terrain—approximately half of the total old town area has slopes exceeding 10-degrees, and 13% of the sampled households are located in such areas. Most residences are accessed via narrow, mixed-traffic streets, where illegal on-street parking further impedes walkability and diminishes pedestrian comfort. By contrast, new towns were developed on predominantly flat terrain and benefit from pedestrian infrastructure and superblocks composed of high-rise apartment complexes, reflecting a planning paradigm that prioritizes vertical density and coordinated spatial organization over compact, ground-level development. This contrast in housing forms illustrates a broader shift in urban planning strategies—from the incremental, low-rise development of old town areas to the vertically oriented and comprehensively planned design of post-1990s new towns.

**Table 2:** *T*-test results comparing objective BE characteristics between old and new towns.

Category		Variables	Old towns	New towns	*t*	Standard error
Pedestrian Environment (within a 400-m network buffer around each respondent’s residence)	Density	Population (persons/400-m buffer)	7127.806	3550.632	−16.212***	220.645
		Household Sample (units/400-m buffer)	1865.805	1133.810	−11.952***	61.247
		Household (households/400-m buffer)	3274.220	1437.875	−17.222***	106.626
	Facility accessibility (POI number/400-m buffer)	Transport facilities	7.648	4.975	−9.132***	0.293
		Shopping centers	0.423	0.370	−0.759	0.069
		Healthcare facilities	4.814	3.753	−1.789	0.593
		Sports service facilities	5.506	3.972	−3.597***	0.426
		Pubs and accommodation facilities	29.099	5.228	−8.722***	2.737
		Community facilities	4.265	2.031	−11.753***	0.190
		Retail facilities	272.264	101.164	−10.503***	16.300
		Schools	0.601	0.466	−1.894	0.071
	Street design	Space Syntax NAIN (average NAIN/400-m buffer)	0.180	0.175	−4.414***	0.001
	Slope	Mean slope within 400-m network buffer of residence	5.365	1.962	−17.248***	0.197
		% households on steep terrain (slopes > 10°)	12.648	0.309	—	—
Park Morphology (within a 400-m network buffer around each respondent’s residence)	Park Shape (1 ha/400-m buffer)	Core area	333.910	334.801	0.05	17.787
		Edge density	21.926	21.147	−0.868	0.897
		Patch Cohesion Index	9.763	9.823	1.464	0.041
		Patch density	0.121	0.127	0.19	0.028
	Park Proximity (km)	Distance to neighborhood park	0.485	0.574	3.842***	0.023
		Distance to small park	0.633	0.414	−8.113***	0.027
		Distance to waterfront park	2.511	0.609	−26.810***	0.071
		Distance to mountain	2.644	2.072	−5.011***	0.114
	Total Park Area (ha)	Neighborhood park	17.723	10.423	−3.402***	2.146
		Small park	0.071	0.384	12.995***	0.024
		Waterfront park	0.261	1.577	5.996***	0.219
		Mountain	7.211	71.380	5.286***	12.140
	In Buffer Park Area (1 ha/400-m buffer)	Neighborhood park	0.817	0.255	−6.180***	0.091
		Small park	0.061	0.215	9.664***	0.016
		Waterfront park	0.023	0.332	7.243***	0.043
		Mountain	0.000	0.049	3.598***	0.014
Sample counts			253	324		

**Significance at 95%; ***significance at 99%.

Differences in block layout and street structure further reinforce this contrast. Old towns are characterized by a fine-grained urban grid with block dimensions typically ranging from 60 m to 100 m. Old towns score marginally higher on road network centrality as measured by Space Syntax NAIN. This configuration supports a highly permeable street network, embedding residential areas in close proximity to commercial facilities. Within a 400-m network, old towns encompass an average of over 200 retail establishments—about three times as many as in new towns—with a greater concentration of leisure and hospitality facilities, such as pubs and accommodations.

By contrast, residential areas in new towns adopt a superblock structure, typically spanning from 200 m to 300 m, that integrates housing, green spaces, and retail functions. Internal green spaces within apartment complexes serve as semi-public areas, whereas retail facilities are typically positioned along the perimeters of superblocks. These residential areas are linked by a town-wide green network that connects neighborhood parks and community facilities. Central business districts are located in designated town centers and are strategically integrated with pedestrian routes and the green network. The street network facilitates separation between pedestrian and vehicular traffic and is designed to support accessibility and safe mobility. Collectively, these planning features contribute to a coherent spatial organization and promote greater integration between residential areas and green infrastructure while enhancing pedestrian connectivity.

The park environment varies substantially between the two areas. Old towns have nearly twice the total area of neighborhood parks compared to new towns, but these are often located on steep terrain or at the urban periphery, limiting routine accessibility. In contrast, new towns contain nearly five times larger small-park areas and six times more waterfront park areas than those in the old town. These parks are more evenly distributed and situated closer to residential areas, with the waterfront park extending through the central area of the new towns. On average, residents in new towns have access to a small park within 0.4 km, and waterfront parks are reachable within a 10-min walk, supporting more frequent use. In contrast, small parks in old towns are typically located over 0.6 km away, and the nearest waterfront park is more than 2.5 km from many homes—approximately four times the distance compared to new towns.

The subjective assessments of the BE aligned with these spatial patterns (Table 3). Residents of new towns reported better satisfaction across multiple domains, including infrastructure quality, natural environment, and access to green spaces. Evaluation of pedestrian environments indicate a favorable perception in new towns, supported by wider sidewalks, appropriate lighting, and dedicated pedestrian routes. Satisfaction with the park environment was also much better among new town residents with respect to entrance clarity, walking-path usability, exercise facilities, and other aspects.

**Table 3:** T-test results comparing perceived BE quality between old and new towns.

**Category**		**Variables**	**Old towns**	**New towns**	**t**	**Standard** **error**
Environmental perception	Perceived neighborhood environment	Parks and greenery	3.097	4.176	14.017***	0.077
		Natural environment (air and water quality)	2.948	3.821	12.282***	0.071
		Living infrastructure (electricity, water/sewage, waste collection)	3.339	4.072	10.092***	0.073
		Public transportation conditions	3.752	4.063	3.955***	0.079
		Medical services	3.523	4.024	6.493***	0.077
		Local public facilities (sports, culture centers, libraries)	3.074	3.764	8.357***	0.083
		Pedestrian environment (slope, sidewalk width, obstacles)	2.807	4.009	14.813***	0.081
		Nighttime lighting and pedestrian safety	3.161	3.940	10.177***	0.077
		Safety from vehicles while walking	2.832	3.722	10.883***	0.082
		Sense of belonging to neighborhood	2.597	3.161	7.048***	0.080
		Perception that neighbors are willing to help	2.610	3.051	5.880***	0.075
		Overall satisfaction with neighborhood	3.184	3.949	11.557***	0.066
	Perceived park environment	Entrances are easy to find	3.698	4.121	6.285***	0.067
		Convenient to reach on foot	3.729	4.326	8.693***	0.069
		Convenient to reach by car (parking)	3.143	3.081	−0.691	0.091
		Walking trails/exercise facilities are easy to use	3.570	3.814	3.483***	0.070
		Outdoor exercise equipment is well maintained	3.330	3.581	3.526***	0.071
		Convenience facilities (toilets, rest areas, benches) are easy to use	3.411	3.677	3.436***	0.077
		Neighborhood facilities around the green space are convenient	3.140	3.540	4.950***	0.081
		Safety management is adequate	3.143	3.581	5.605***	0.078
		There is abundant shade	3.426	3.658	3.011***	0.077
		Overall satisfaction with park	3.531	3.994	7.599***	0.061
Sample counts			253	324		

**Significance at 95%; ***significance at 99%.

In summary, old towns offer a dense, amenity-rich urban fabric that reflects a historically layered development pattern. Although they present certain structural challenges in walkability and green space access, the compact form supports proximity to diverse services. New towns, shaped by comprehensive master planning, provide well-connected pedestrian networks and strategically located parks, foster daily PA and enhance overall satisfaction with the residential environment.

### 3.2 Comparison of resident characteristics and park usage between old and new towns

The contrasting development strategies of old and new towns in Seongnam City are reflected in both the socioeconomic profiles of residents and their park usage behaviors. As shown in Table 4, residents of new towns reported higher levels of income and education, which is consistent with the planned development of these areas to accommodate middle and upper-middle class populations. By contrast, old towns, shaped by incremental and small-scale development and a higher prevalence of low-rise multi-family dwellings, support a socioeconomically diverse population.

**Table 4:** Socioeconomic status between old and new towns.

Category		Variables	Old towns	New towns	Chi-square
Demographic characteristics	Age	Youth (15–29 years)	26.877%	22.531%	4.269
		Middle-aged (30–49 years)	62.055%	60.802%	
		Older (50–69 years)	11.067%	16.667%	
	Income	Low income (less than 200 thousand won)	16.206%	16.049%	26.920***
		Middle income (200–500 thousand won)	73.123%	56.173%	
		High income (more than 500 thousand won)	10.672%	27.778%	
	Gender	Male	35.968%	32.716%	0.668
		Female	64.032%	67.284%	
	Education	College degree or higher	78.656%	93.210%	26.311***
		High school or below	21.344%	6.790%	
	Housing type	High-rise apartment complex	41.107%	83.025%	108.087***
		Low-rise multi-family housing	45.850%	16.049%	
		Single-family housing	13.043%	0.926%	
Sample counts			253	324	

**Significance at 95%; ***significance at 99%.

**Figure 4:**
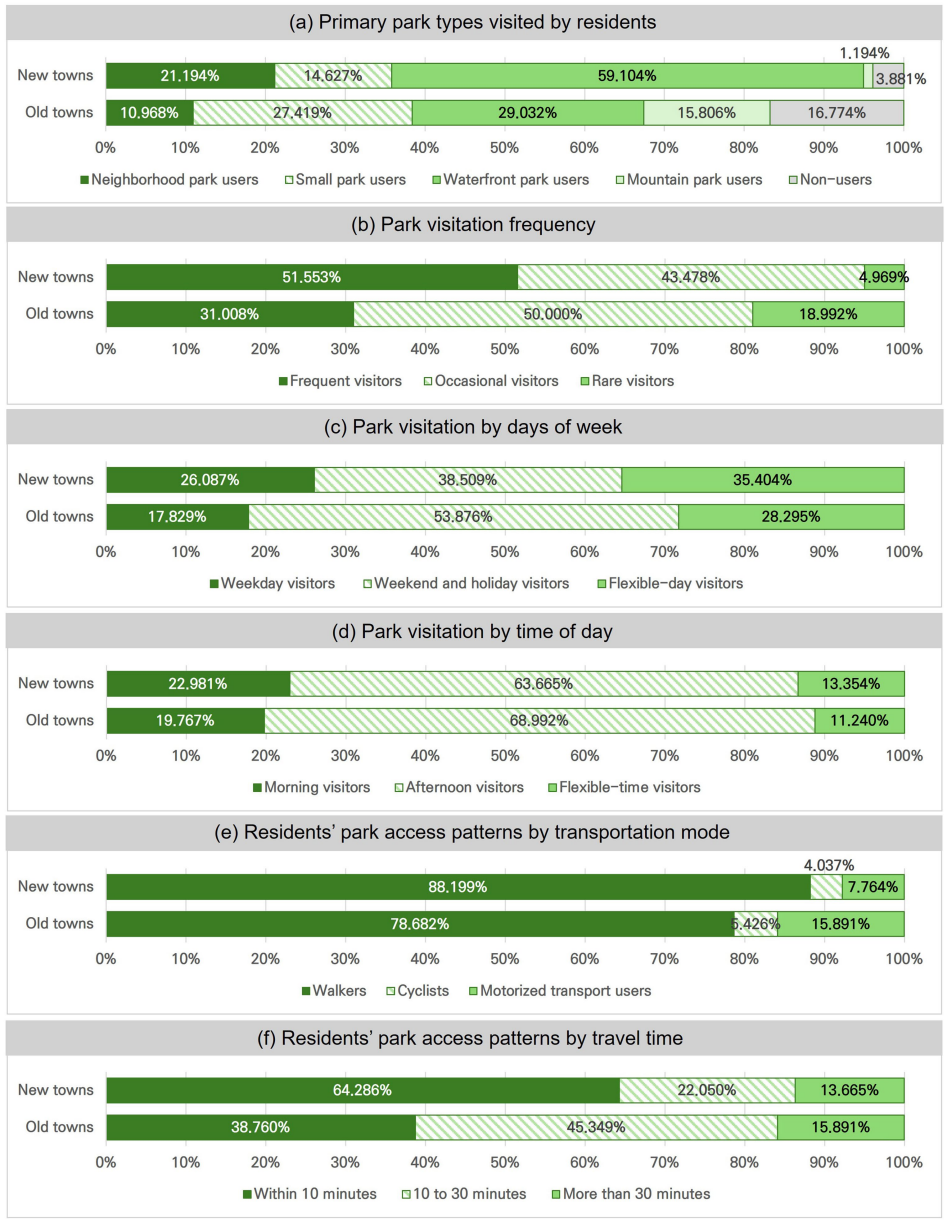
Differences in park usage between old and new towns. **(a)** Primary park types visited by residents. **(b)** Park visitation frequency. **(c)** Park visitation by days of the week. **(d)** Park visitation by time of day. **(e)** Residents’ park access patterns by transportation mode. **(f)** Residents’ park access patterns by travel time.

Preferences for park types varied notably between the two areas, reflecting differences in spatial integration and accessibility (Figure 4). In new towns, 60% of residents primarily used the centrally located waterfront park, which is embedded within a connected green network originating from residential superblocks. This was followed by 21% who preferred neighborhood parks and 15% who used small parks. Only 4% of new town residents reported not using any parks, indicating widespread and routine engagement with nearby green space. In contrast, old town residents exhibited a more dispersed pattern of park use: 29% used the waterfront park, 27% preferred small parks, and 16% visited mountain parks. Both waterfront and mountain parks in old towns are situated more than average 2 km from residential areas and are less conducive to routine visits. Nearly 17% of old town residents reported not using any parks—over four times the proportion observed in new towns.

Park usage frequency also differed between two towns. In new towns, 52% of residents were frequent users who visited parks daily or two to three times per week, while 43% were occasional users visiting once or twice a month. In contrast, only 31% of old town residents were frequent users, while about half used parks infrequently, and nearly one-fifth reported no park use at all. This discrepancy underscores how both park availability critically shape not only access but also residents’ preferences and usage behaviors.

Temporal patterns also differed: new town residents predominantly use parks during weekday afternoons, whereas old town residents tend to visit on weekends or holidays. In new towns, most residents access parks on foot, within 10 min, reflecting strong pedestrian connectivity and deliberate proximity in design. In old towns, longer travel times and greater reliance on public transport or private vehicles indicate lower walkability and less evenly distributed park networks.

Health-related indicators showed modest variations across the two urban contexts (Table 5). Old town residents reported slightly higher self-rated health-related quality of life(EQ-5D), but differences were neither statistically significant nor clinically meaningful. PA levels(IPAQ MET values) remained similar across both groups. The absence of substantial baseline differences reinforces the need for a multivariable regression analysis to identify the specific contribution of park morphology to activity levels.

**Table 5:** Health characteristics between old and new towns.

**Variables**		**Old towns**	**New towns**	**t**	**Standard error**
Level of PA	IPAQ-MET	3471.114	3042.553	−1.244	344.371
Health characteristics (EQ-5D)	EQ-5D Index	0.805	0.792	−0.723	0.017
	- Mobility	1.767	1.883	1.301	0.089
	- Self-Care	1.328	1.355	0.418	0.064
	- Usual Activities	1.522	1.580	0.748	0.078
	- Pain/Discomfort	1.885	1.880	−0.063	0.092
	- Anxiety/Depression	1.826	1.827	0.012	0.090
**Sample counts**		253	324		

**Significance at 95%; ***significance at 99%. The EQ-5D Index ranges up to 1, with higher values indicating better health status. Each sub-dimension (Mobility, Self-care, Usual Activities, Pain/Discomfort, Anxiety/Depression) is measured on a 1 (no problems) to 5 (extreme problems) scale. Higher scores in the sub-dimensions reflect poor health and correspond to lower EQ-5D Index values.

### 3.3 Determinants of PA across contrasting urban contexts (logistic regression findings)

A municipality typology variable (new towns = 1, old towns = 0) was included to control for structural differences, including the significant disparities in mean slope and waterfront access documented in Table 2, and isolate independent effects of park morphology. To minimize multicollinearity, we adopted a conservative threshold of VIF < 5 for variable inclusion. Following this criterion, several indicators with high redundancy, such as population and retail densities, were excluded. Consequently, the final regression models achieved high stability, with all remaining predictors maintaining VIF values below 1.9 (e.g., maximum VIF = 1.801), ensuring that the estimated effects of park morphology are robust. As confirmed by prior t-tests, most BE indicators differed significantly between the old and new towns, justifying the use of the typology variable. Interaction terms were also included to assess if the influence of park morphology varied by urban context and its effects were specific to certain spatial settings.

The ‘City-wide’ model includes the entire sample (n = 577) and establishes the baseline association between BE factors and PA for Seongnam City as a whole. Its primary purpose is to allow for the subsequent analysis of interaction terms in the ‘City-wide (Interaction)’ model. The significance of these interaction terms confirms the central finding that the influence of park morphology on PA is context-specific and varies significantly between old and new towns.

Within Table 6, distinct sociodemographic patterns emerged between the old and new towns. In old towns, gender and education were strong PA predictors. Females had approximately 68% lower odds of being active, while university graduates were over three times more likely to be active than those with high school education or below. In new towns, education remained significant with university graduates showing 3.5 times greater odds of being active. High-income residents were more likely to be active in new towns but less active in old towns compared to middle-income residents. Youth(15–29 years) showed higher activity likelihood in new towns, and better self-rated health (EQ-5D index) increased PA odds over seven times in new towns.

**Table 6:** Binary logistic regression analysis for old towns, new towns, and city-wide (entire towns).

Variables			Old towns	New towns	City-wide	City-wide (Interaction)
			Exp(B)	Exp(B)	Exp(B)	Exp(B)
Personal characteristics	Age	Middle-aged (30–49 years)	*References*			
		Youth (15–29 years)	1.347	2.641*	1.566	1.574
		Older (50–69 years)	2.677	1.030	1.262	1.277
	Gender	Gender (Male = 0)	*References*			
		Gender (Female = 1)	0.323**	0.648	0.475**	0.463**
	Income	Middle income (200–500 thousand won)	*References*			
		Low income (less than 200 thousand won)	1.305	1.523	1.576	1.491
		High income (more than 500 thousand won)	0.252**	2.431*	1.000	0.967
	Education	Education (high school or below = 0)	*References*			
		Education (college degree or higher = 1)	3.164**	3.470**	2.510**	2.513**
	Perceived health	Health Related Quality of Life (EQ-5D index)	2.576	7.232**	3.516**	3.559**
Park utilization and perception	Park visit frequency	Rare visit (less than once a month)	*References*			
		Occasional visit (once a week, 1–2 times a month)	4.285***	0.494	1.642	1.812*
		Frequent visit (daily, 2–3 times a week)	7.248***	3.098	5.654***	6.502***
	Perceived park environment	Park near convenience facilities	2.287***	1.159	1.404**	1.374*
		Park exercise Equipment	0.735	0.703	0.789	0.769
		Park safety management	0.714	0.753	0.768	0.773
BE characteristics	Municipal typology	Old town = 0	*References*			
		New town = 1			0.954	0.116**
	Facility accessibility	Community facilities	1.212*	1.196	1.213***	1.180**
		Pub and accommodation facilities	0.991*	0.994	0.994	0.994*
	Park shape	Edge Density	0.988	1.067**	1.009	0.991
	Park area	In-Buffer Park Area_Neighborhood Park	0.882	10.144**	1.128	0.981
		Total Park Area_Mountain	1.046	1.000	1.001	1.001
	Interaction (Park Morphology × Municipal Typology)	Edge Density * Municipal Typology				1.083**
		In-Buffer Park Area_Neighborhood Park * Municipal Typology				8.319**
*n*			253	324	577	577
Constant			0.704	0.347	0.732	1.500
−2 Log Likelihood			161.370	189.257	389.361	374.229
Cox & Snell ~ Nagelkekerke R square			0.165 ~ 0.295	0.129 ~ 0.251	0.087 ~ 0.162	0.111 ~ 0.207

*Significance at 90%; **significance at 95%; ***significance at 99%. Odds ratios are reported in Table 6. Corresponding 95% confidence intervals for all models are provided in Appendix Table S2.

Built environment features showed markedly different associations with physical activity in the old and new towns. In new towns, spatial design variables play a prominent role. Edge density, which captures spatial complexity, reflects the extent of park connectivity and the accessibility of park boundaries within each respondent’s 400-m walkable network. This metric was significantly associated with physical activity, corresponding to approximately a 7% increase in the probability of participation. Similarly, an additional hectare of neighborhood parks within walking distance was associated with a tenfold increase in the likelihood of engaging in physical activity. These results underscore the efficacy of integrated green infrastructure and coherent spatial planning in promoting routine physical activity. By contrast, in old towns, none of the spatial park metrics, including edge density and park area, were associated with activity. The limited impact of BE features in these settings may reflect fragmented park distribution, topological barriers, and a high concentration of nightlife establishments such as pubs, bars, and motels, and the absence of coordinated planning. Collectively, these factors hamper the functional integration of parks into residents’ daily routines. However, the availability of community facilities in old towns showed a positive association with physical activity, underscoring the importance of accessible, non-commercial social infrastructure in compensating for structural limitations in the urban fabric.

Park usage patterns revealed varying associations with PA between the old and new towns. In old towns where parks are spatially less integrated, visit frequency is a significant predictor of activity. Occasional visits (1–2 times per month) were associated with over four times higher odds of being active, whereas frequent visits (2–3 times per week or daily) showed nearly seven times higher odds. By contrast, visit frequency did not show a statistically significant association with activity in new towns. This difference reflects the variation in how park use is linked to activity based on the urban context. Perception-based variables demonstrated limited influence in both areas. Satisfaction with convenience facilities near parks had a modest impact on the citywide model, whereas other perception measures such as park safety and the presence of exercise equipment were not significantly associated with activity.

The interaction terms further confirmed that spatial design attributes have a context-specific effect on PA. Edge density was significantly associated with higher odds of activity in new towns, signifying that conditional influence in the presence of a coordinated urban structure that facilitates connectivity between parks and surrounding pedestrian networks. However, this association was not observed in old towns, where urban form was fragmented and systematically less integrated. The positive relationship between neighborhood park areas and activity was statistically significant in the context of only the new town. These results indicate that spatial park metrics, such as edge density and park area, contribute to PA outcomes when supported by a planning framework that ensures accessibility and functional integration within the BE.

## 4 Discussion

### 4.1 Spatial structure and green space equity across suburban contexts

Green spaces equity entails not only the physical provision of parks but also the extent to which these spaces are usable, accessible, and integrated into residents’ daily lives. In Seongnam, marked differences in park usage patterns between old and new towns reveal how spatial structure mediates functional equity. Although old towns have less total park overall, they contain a larger total area of neighborhood parks compared to new towns. However, many of these are situated on steep hillsides or at the urban fringe, limiting their accessibility. The covering of a stream that once flowed through the town center during 1990s redevelopment has further reduced access to centrally located green space. Correspondingly, old town residents were more likely to visit parks infrequently, typically on weekends or holidays, and often relied on public transportation or private vehicles due to long travel distances and fragmented pedestrian networks.

By contrast, new towns with systematically planned spatial layouts promote more frequent use of green spaces. Small neighborhood parks and green pockets are evenly distributed within high-density residential superblocks and are directly connected through walkable paths and green corridors. Especially, a centrally located waterfront park functions as a linear spine passing through the residential core, creating multiple proximate entry points and linking local pockets into a continuous network. This spatial configuration enables most residents to access parks on foot within 10 min, contributing to higher daily or weekly use, particularly during weekday afternoons. These behavioral differences underscore that equitable access is not simply a matter of park quantity but also of their spatial configuration, proximity to homes, and ease of access (9, 48).

Differences in perceived BEs further underscore spatial inequities between old and new towns. Residents of new towns expressed significantly higher satisfaction across key domains, including access to park, infrastructure quality, pedestrian safety, and a stronger sense of neighborhood belonging. These perceptions are supported by the presence of well-connected sidewalks, sufficient lighting, and user-friendly park features such as clearly marked entrances and convenient walkable access. In contrast, residents of old towns reported lower satisfaction with both neighborhood and park environments, reflecting barriers such as fragmented street networks, steep terrain, and limited pedestrian infrastructure. These perceptual disparities highlight that park equity extends beyond physical provision to encompass the quality and usability of the surrounding environment, which directly influences residents’ willingness and ability to engage with nearby parks. As such, integrating physical design considerations into suburban planning is essential for addressing spatial inequities and advancing broader goals of public health and environmental justice. Ultimately, our results reveal that the BE can either act as a barrier or a catalyst for health equity. In old towns, poor environmental quality acts as a ‘behavioral tax’ that disproportionately suppresses the activity of women and high-income residents. Conversely, the integrated structure of new towns acts as a ‘behavioral enabler’, neutralizing individual socio-demographic constraints and enabling health intentions to be seamlessly translated into physical action.

### 4.2 Role of park morphology in promoting PA

The findings from Seongnam City highlight the critical role of park morphology, particularly park configuration and edge articulation, in shaping PA within suburban environments. In new towns where pedestrian-oriented planning supports routine engagement with green infrastructure, morphological features are strongly associated with increased activity levels. A 1-m increase in edge density per hectare, indicating more articulated and accessible park boundaries, corresponded to a 7% increase in the odds of being physically active. Moreover, each additional hectare of a neighborhood park within a 400-m buffer was associated with a more than tenfold increase in the likelihood of meeting the recommended 600 MET minutes of weekly activity. These findings align with prior research that design interventions, such as looped walking paths and edge-rich linear park layout scan, enhance PA substantially (49, 50).

This study shows that the impact of park morphology is not uniform across contexts. In old towns, where green spaces are spatially disconnected, access to linear green spaces such as waterfront parks is limited. Moreover, factors such as steep slopes, a high concentration of nightlife facilities, and mixed-traffic conditions persist. As a results, spatial configuration variables—including edge density and park area—are not significantly associated with PA. Rather, the frequency of park visits emerged as the primary behavioral correlation, suggesting that PA in these settings depends on discretionary travel behavior than the park form.

These patterns highlight a broader planning challenge. The presence of green spaces does not guarantee or promote PA if the surrounding urban form fails to support access and engagement. This inference aligns with Western contexts, where disparities in park use and health outcomes persist despite comparable levels of green space provision (51, 52). In Seongnam’s old towns, being female or belonging to a high-income group was associated with lower odds of PA (three to four times lower), signifying that structural constraints in the BE may disproportionately affect certain groups. Conversely, in new towns, gender did not affect PA levels, and high-income residents were more likely to be active, indicating a more enabling urban context. These findings highlight that engagement with green spaces is shaped by individual sociodemographic factors and the degree to which the BE facilitates routine and equitable access. Accordingly, morphological improvements to parks will be most effective when integrated with broader efforts to enhance walkability, strengthen pedestrian infrastructure, and address spatial inequities across neighborhood contexts.

These findings emphasize the importance of adopting context-sensitive strategies while planning green spaces to reflect the varying influences of spatial design across different urban settings. In newly developed areas, where pedestrian-oriented planning ensures spatial coherence and accessibility, maintaining and enhancing morphological attributes such as articulated park edges, adequate provision of neighborhood parks, and integration of community facilities can further enhance the health-promoting role of green infrastructure. The strong associations observed in new towns between park edge density, neighborhood park area, and activity levels supports prioritizing and incorporating such design features into future planning. As proximity is addressed in new towns, planners should focus on enhancing diversity and continuity. Lengthening greenway loops through superblocks, varying vegetation structure, and programming seasonal events will ensure that residents do not become habituated to a limited set of stimuli. Finally, increased density of community facilities can be a universal booster, and co-locating cultural centers or cafés at park edges can turn single-purpose trips into multifaceted, active journeys.

By contrast, old town areas characterized by steep slopes, limited connectivity to linear parks, and insufficient pedestrian infrastructure, along with high concentrations of nightlife facilities such as pubs, bars, and motels, present considerable barriers to walkability. These features generate environmental stressors including noise, crowding, and perceived safety concerns, which collectively diminish the comfort and attractiveness of walking. In such environments, improvements to the morphology of parks alone may be insufficient to promote active living, as the broader urban context remains unsupportive.

Given the complex constraints observed in old town areas, interventions must transcend conventional park redesign efforts. Enhancing walkability by improving sidewalk connectivity and lighting can mitigate perceived and actual safety concerns. In addition, the strategic distribution of small, accessible green pockets near residential clusters may offer more practical benefits than large, peripheral parks that are difficult to access. Another feasible approach involves the partial restoration of Dandae Stream that was covered during 1990s redevelopment and now serves as a major roadway. While full restoration may not be realistic, selectively reopening sections—paired with traffic calming measures—could reintroduce valuable green space and create linear park elements that improve accessibility and passive recreational use.

Complementing these physical interventions, the observed positive association between community facilities and PA underscores the importance of accessible, non-commercial social infrastructure—such as libraries, neighborhood welfare centers, and senior community centers—in offsetting the structural limitations of the BE. These facilities may serve as key anchors in residents’ daily routines, supporting incidental PA and fostering neighborhood cohesion. Furthermore, as park use in old town areas often depends more on individual initiatives than environmental support, social interventions that promote health literacy and encourage community engagement may be necessary to translate spatial proximity into regular use. Overall, the findings suggest that maximizing the health benefits of green spaces requires a coordinated planning approach that combines morphological improvements with broader efforts to address the structural and behavioral barriers to park use.

### 4.3 Study implications and limitations

This study contributes to the growing body of research examining the relationship between urban form, park morphology, and PA, offering several key implications for urban planning and public health. By employing high-resolution spatial indicators and a validated instrument for PA measurement, it improves the methodological precision of assessing how park characteristics influence behavioral outcomes. The use of standardized and geocoded indicators strengthens the comparability and reliability of findings, particularly in suburban settings where spatial variability is often underexplored.

The comparative analysis between master-planned new towns and incrementally developed old towns within the same municipal boundary presents a unique opportunity to isolate the effects of planning legacies on active living. This intra-city design controls for broader regional variation and highlights how urban form and spatial context—beyond total green space provision—shapes opportunities for routine PA. Notably, the findings demonstrate that spatial integration, pedestrian infrastructure, and the morphological features of parks interact with sociodemographic factors to influence both park use and activity levels. This underscores the importance of tailoring green space interventions to distinct neighborhood typologies, where physical form, infrastructure deficits, and social conditions interact in complex ways to either hinder or support active living. From a planning perspective, the study reinforces the necessity of aligning physical design with broader goals of environmental justice, ensuring that all residents—regardless of income or location—have equitable access to health-promoting environments.

Despite these contributions, several limitations should be acknowledged. First, PA and environmental perceptions were measured through self-reported survey data, which may be affected by subjective bias or temporary mood states. Although validated instruments were used, the possibility of over- or underreporting cannot be ruled out. Second, the cross-sectional design limits the ability to draw causal inferences. While the analysis identified associations between park features and activity levels, it remains unclear whether specific morphological attributes promote activity or whether more active individuals choose to live in better-served environments. Third, the geographical proximity of the two study sites may allow for ‘cross-over usage’ of infrastructure, where residents travel beyond their immediate neighborhood buffers to utilize superior park facilities in adjacent areas. While such fluid movement patterns could moderate the influence of the immediate residential environment, this study primarily focuses on home-based buffers to examine how local environmental affordances drive routine, daily physical activity. Fourth, although visitation frequency was included, the study may not have fully captured short, incidental, or low-intensity park visits that are often overlooked in standard survey formats but may still be influenced by spatial features such as high edge density or visual permeability. Finally, the study is geographically limited to a single municipality. While the contrast between old and new towns within the same suburban city enhances internal validity by controlling for contextual differences, this narrow scope may limit the generalizability of the findings to other urban settings with different spatial and cultural dynamics.

Future research would benefit from longitudinal or quasi-experimental designs that evaluate changes in PA and park use before and after green space improvements. Incorporating objective measures such as GPS tracking, accelerometry, and time-use data could also enhance the accuracy and depth of behavioral assessments. Comparative studies across multiple cities with varying urban typologies could further validate the findings and inform context-sensitive strategies for integrating green infrastructure into suburban planning. Ultimately, aligning spatial data with behavioral outcomes remains essential for designing equitable, health-promoting urban environments.

## 5 Conclusion

This study underscores the importance of spatial form in shaping suburban residents’ interaction with green spaces, offering new evidence that the relationship between park morphology and PA is deeply context-dependent. By integrating high-resolution morphological metrics with behavioral data across differently planned suburban areas, this study highlights that spatial design can either enable or constrain routine park use. In newly developed areas with coherent urban forms, features such as edge density, park size, and facility integration support PA. However, in older neighborhoods with topographical and infrastructural limitations, the same morphological features yield weaker outcomes, suggesting that behavioral engagement depends on discretionary choices and broader environmental support.

Taken together, these results suggest the need to move beyond uniform park provision models toward differentiated planning strategies that respond to local structural conditions. Green space should not be viewed solely as a fixed quantity, but rather as a dynamic element whose value depends on how it is spatially embedded and socially accessible. Spatial legacies and neighborhood form fundamentally shape who benefits from green spaces, how they are used, and whether they support daily opportunities for PA, informal social interaction, and restorative experiences that contribute to overall well-being. As Wolch et al. (9) argued, urban greening should not only expand coverage but also correct historical inequities and functional inaccessibility. This study reinforces this call by emphasizing that green infrastructure must be embedded in everyday urban life through both spatial and social mechanisms. Future planning for healthy, equitable cities will benefit from integrating park morphology into broader urban design systems that support inclusive, sustained PA.

## Data Availability

The datasets presented in this article are not readily available because the dataset contains human-subject survey and spatial/health information. IRB and ethical approval do not allow sharing this dataset outside the research team. Requests to access the datasets should be directed to jeongwoo@cau.ac.kr.
