# Supplementary material for: Park morphology and urban structure for active living: a suburban case from Seongnam City
Source: Front Public Health. 2026 Jan 23;14:1744227. doi: 10.3389/fpubh.2026.1744227 (PMC12876230; doi:10.3389/fpubh.2026.1744227)
Supplement: Supplementary file 1 [file Supplementary_file_1.docx]

**Appendix**

**Table S1** Sensitivity Analysis of the Relationship Between Physical Activity (IPAQ) and Environmental Indices Across Different Catchment Radii

| **Sensitivity Parameter**  **(Catchment Radius)** | **Coefficient**  **(Effect on IPAQ)** | **P-value** | **95% Confidence Interval** |
| --- | --- | --- | --- |
| 400m | .097** | .004 | [0.031, 0.162] |
| 500m | .085* | .012 | [0.012, 0.018] |
| 600m | .087** | .009 | [0.021, 0.151] |
| 700m | .081* | .014 | [0.016, 0.146] |
| 800m | .071* | .036 | [0.004, 0.134] |

**Figure S1** Sensitivity Analysis Graph of Catchment Radii


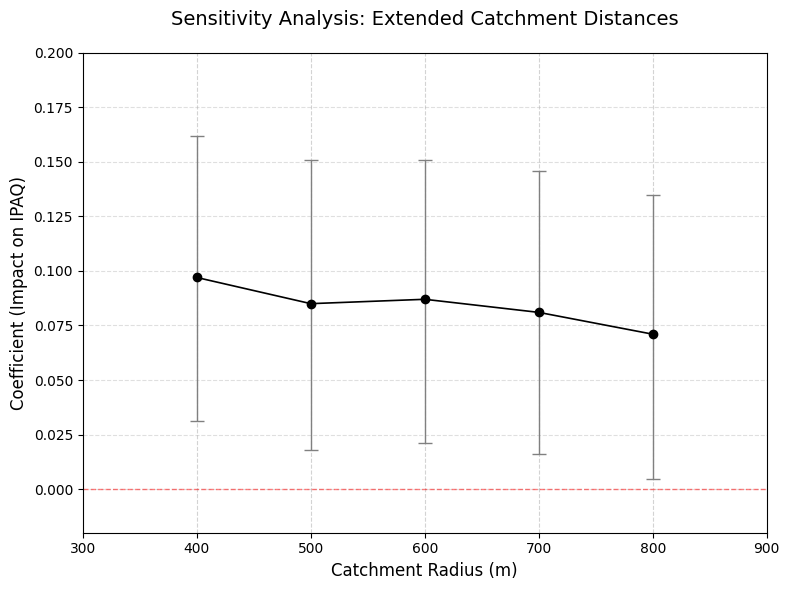


Table S2 Odds Ratios and 95% Confidence Intervals for the Binary Logistic Regression Models

| **Variables** | **Old Towns (n=253)** | **New Towns (n=324)** | **City-wide (n=577)** | **City-wide (Interaction)** |
| --- | --- | --- | --- | --- |
| **Personal Characteristics** | **Exp(B) [95% CI]** | **Exp(B) [95% CI]** | **Exp(B) [95% CI]** | **Exp(B) [95% CI]** |
| Age: Youth (15–29) | 1.347 [0.499, 3.640] | 2.641 [0.926, 7.531] | 1.566 [0.807, 3.038] | 1.573 [0.803, 3.083] |
| Age: Older (50–69) | 2.677 [0.578, 12.397] | 1.030 [0.344, 3.087] | 1.262 [0.547, 2.911] | 1.277 [0.547, 2.982] |
| Gender: Female | 0.323 [0.118, 0.881] | 0.648 [0.273, 1.535] | 0.475 [0.255, 0.882] | 0.463 [0.246, 0.870] |
| Income: Low income | 1.305 [0.410, 4.156] | 1.523 [0.462, 5.020] | 1.576 [0.721, 3.443] | 1.461 [0.662, 3.224] |
| Income: High income | 0.252 [0.072, 0.885] | 2.431 [0.897, 6.589] | 1.000 [0.485, 2.064] | 0.967 [0.463, 2.021] |
| Education: College or higher | 3.164 [1.227, 8.156] | 3.470 [0.832, 14.474] | 2.510 [1.227, 5.134] | 2.513 [1.214, 5.202] |
| Health Quality (EQ-5D) | 2.576 [0.391, 16.993] | 7.232 [1.126, 46.443] | 3.516 [1.067, 11.588] | 3.559 [1.040, 12.173] |
| **Park Utilization** |  |  |  |  |
| Occasional visit | 4.285 [1.622, 11.321] | 0.494 [0.120, 2.034] | 1.642 [0.860, 3.134] | 1.812 [0.928, 3.537] |
| Frequent visit | 7.248 [1.650, 31.832] | 3.098 [0.689, 13.927] | 5.654 [2.414, 13.243] | 6.502 [2.686, 15.737] |
| Near convenience fac. | 2.287 [1.228, 4.258] | 1.159 [0.729, 1.841] | 1.404 [1.005, 1.961] | 1.374 [0.974, 1.939] |
| Park exercise equipment | 0.735 [0.368, 1.468] | 0.703 [0.394, 1.256] | 0.789 [0.529, 1.177] | 0.769 [0.507, 1.168] |
| Park safety management | 0.714 [0.333, 1.531] | 0.753 [0.435, 1.302] | 0.768 [0.516, 1.144] | 0.773 [0.508, 1.178] |
| **BE Characteristics** |  |  |  |  |
| Municipal Typology |  |  | 0.954 [0.475, 1.915] | 0.116 [0.027, 0.507] |
| Community facilities | 1.212 [1.025, 1.434] | 1.196 [0.857, 1.670] | 1.213 [1.056, 1.393] | 1.180 [1.026, 1.357] |
| Pub/Accommodation fac. | 0.991 [0.982, 0.999] | 0.994 [0.987, 1.002] | 0.994 [0.987, 1.002] | 0.994 [0.986, 1.001] |
| Edge Density | 0.988 [0.963, 1.014] | 1.067 [1.008, 1.129] | 1.009 [0.981, 1.038] | 0.991 [0.967, 1.016] |
| In-Buffer Park Area | 0.882 [0.658, 1.182] | 10.144 [1.530, 67.265] | 1.128 [0.839, 1.518] | 0.981 [0.737, 1.306] |
| Total Park Area (Mountain) | 1.046 [-] | 1.000 [0.998, 1.003] | 1.001 [0.999, 1.003] | 1.001 [0.999, 1.003] |
| **Interaction Terms** |  |  |  |  |
| Edge Density × Typology | - | - | - | 1.083 [1.019, 1.151] |
| Park Area × Typology | - | - | - | 8.319 [1.379, 50.181] |
